# Genetic variation and association of yield, yield components, and carbon storage in sorghum (*Sorghum bicolor* [L.] Moench) genotypes

**DOI:** 10.1186/s12863-024-01256-4

**Published:** 2024-08-01

**Authors:** Asande Ngidi, Hussein Shimelis, Seltene Abady, Vincent Chaplot, Sandiswa Figlan

**Affiliations:** 1 African Centre for Crop Improvement, School of Agricultural, Earth and Environmental Sciences, University of KwaZulu-Natal, Private Bag X01, Scottsville, Pietermaritzburg, 3209 South Africa; 2 School of Agricultural, Earth and Environmental Sciences, University of KwaZulu-Natal, Private Bag X01, Scottsville, Pietermaritzburg, 3209 South Africa; 3 Laboratory of Oceanography and Climate, Experiments and Numerical Approaches (LOCEAN), UMR 7159, IRD/C NRS/UPMC/MNHN, IPSL, Paris, 75005 France; 4 Department of Agriculture and Animal Health, University of South Africa, Florida, 1709 South Africa

**Keywords:** Carbon sequestration, Genetic advance, Genetic variability, Broad-sense heritability, Organic carbon, Sorghum

## Abstract

Trait heritability and the response to selection depend on genetic variation, a prerequisite to developing sorghum varieties with desirable agronomic traits and high carbon sequestration for sustainable crop production and soil health. The present study aimed to assess the extent of genetic variability and associations among agronomic and carbon storage traits in selected sorghum genotypes to identify the best candidates for production or breeding. Fifty genotypes were evaluated at Ukulinga, Bethlehem and Silverton sites in South Africa during the 2022/23 growing season. The following agronomic and carbon storage traits were collected: days to 50% heading (DTH), days to 50% maturity (DTM), plant height (PH), total plant biomass (PB), shoot biomass (SB), root biomass (RB), root-to-shoot biomass ratio (RS), grain yield (GY), harvest index (HI), shoot carbon content (SCc), root carbon content (RCc), grain carbon content (GCc), total plant carbon stock (PCs), shoot carbon stock (SCs), root carbon stock (RCs), and root-to-shoot carbon stock ratio (RCs/SCs), and grain carbon stock (GCs). Higher genotypic coefficient of variations (GCVs) were recorded for GY at 45.92%, RB (39.24%), RCs/SCs (38.45), and RCs (34.62). Higher phenotypic coefficient of variations (PCVs) were recorded for PH (68.91%), followed by GY (51.8%), RB (50.51%), RS (41.96%), RCs/SCs (44.90%), and GCs (41.90%). High broad-sense heritability and genetic advance were recorded for HI (83.76 and 24.53%), GY (78.59 and 9.98%), PB (74.14 and 13.18%) and PCs (53.63 and 37.57%), respectively, suggesting a marked genetic contribution to the traits. Grain yield exhibited positive association with HI (*r* = 0.76; *r* = 0.79), DTH (*r* = 0.13; *r* = 0.31), PH (*r* = 0.1; *r* = 0.27), PB (*r* = 0.01; *r* = 0.02), RB (*r* = 0.05; *r* = 0.06) based on genotypic and phenotypic correlations, respectively. Further, the path analysis revealed significant positive direct effects of SB (0.607) and RB (0.456) on GY. The RS exerted a positive and significant indirect effect (0.229) on grain yield through SB. The study revealed that PB, SB, RB, RS, RCs, and RCs/SCs are the principal traits when selecting sorghum genotypes with high yield and carbon storage capacity.

**Supplementary Information:**

The online version contains supplementary material available at 10.1186/s12863-024-01256-4.

## Introduction

Sorghum (*Sorghum bicolor* [L.] Moench) is a resilient crop adapted to grow in diverse agro-ecologies, including semi-arid, subtropical, tropical and temperate climates globally. It is a primary staple food crop for most of Africa’s semi-arid regions, including South Africa. It thrives under drought conditions where other major cereal crops fail [1–3]. Sorghum seeds are rich sources of nutrients such as carbohydrates (65–76%), proteins (8–12%), dietary fibre (2%), vitamin B, and minerals (e.g. iron, magnesium, phosphorus, and potassium) [4]. Sorghum stover contains crude protein (8–12%), digestible organic matter and metabolizable energy (70–75%) [5]. Sorghum’s nutritional content and resilience to grow under harsh growing conditions make it an ideal dual-purpose crop in a marginal and mixed livestock-cropping system.

Sorghum has a C4 photosynthetic pathway with high photosynthesis efficiency and biomass production which are vital under limited water and soil nutrients facilitated by its extended root length, density, and water-use efficiency. It can reach a height of 4 m and produce fresh biomass yields ranging from 45 to 112 tons per hectare, depending on genotype and growing environments [6]. The high biomass production potential of sorghum contributes to increased carbon sequestration by efficiently capturing and storing atmospheric carbon dioxide (CO_2_) through photosynthesis, reducing and compensating for emissions.

Druille et al. [7] reported that changes in plant carbon allocation patterns due to climate change can significantly impact agricultural productivity and food security. Crop genetic resources with adequate genetic variation are vital to developing new cultivars with desirable agronomic traits and high carbon sequestration for sustainable crop production and soil health. Phenotypic variation depends on the test population’s genetic constitution and the growing conditions.

Continued development of sorghum cultivars is crucial to mitigate future climate change impacts and sustainably feed a growing global population. This can be accomplished by effectively selecting genetically superior and resilient genotypes based on the degree of diversity in the source material. The success of any crop improvement relies on the extent of genetic variability present in the source material and the effectiveness of selection [8]. The genotypic coefficient of variation (GCV) and phenotypic coefficient of variation (PCV) provide information on the relative degree of phenotypic and genotypic variation in distinct traits of sampled populations [9]. Heritability is predictive in indicating the reliability of phenotypic value as a guide to breeding value [10]. High values of heritability indicate that the phenotypic expression of the genotype is a good indicator of its genetic potential. The degree of genetic gain acquired in an economic trait under a specific selection pressure is explained by genetic advance. Selection progress depends on genetic variability for yield and yield contributing traits and their heritability [11]. High genetic advance and heritability estimates demonstrate the genetic effects of conditioning economic trait, indicating the efficiency of selection in crop improvement programs [12].

Correlation analysis provides information on the degree of relationship between essential traits. It can facilitate the selection and improvement of complex polygenic traits, including grain yield in sorghum. Understanding the extent of relationship between yield and yield components is crucial to improving selection efficiency for high yields [13]. Correlations between agronomic traits and high heritability simplify the selection process for complex traits like grain yield [14]. Consequently, opting for agronomic traits with strong correlations can increase genetic gains. Selection based on phenotypic performance may not result in expected genetic advancement due to genotype-by-environment interactions. In this regard, A multi-location and -seasonal assessment of sorghum genotypes is essential to develop the best-performing varieties for yield components and carbon storage. Path coefficient analysis is used to partition the correlation between yield and component traits into direct and indirect effects, identify the cause-effect relationship, and devise effective selection strategies [15]. Genetically diverse sorghum accessions were collected from major producing countries, including Ethiopia, Tanzania, and South Africa, for selection under South African conditions. From this gene pool, 50 accessions were sampled based on their high grain yield, biomass, and ethanol production [16]. The objective of this study was to assess the extent of genetic variability and associations among agronomic and carbon storage traits in selected genetically diverse sorghum genotypes to select contrasting traits for production or breeding.

## Materials and methods

### Plant material, trail design and management

Fifty sorghum genotypes obtained from South Africa (Agricultural Research Council, African Centre for Crop Improvement and Department of Agriculture), Zimbabwe, Ethiopia, and Tanzania were used in this study (Table 1). The genotypes were primarily selected based on their high grain yield, biomass, and ethanol production [16]. The 50 genotypes were evaluated in three environments: Ukulinga research farm at the University of KwaZulu-Natal in Pietermaritzburg, Bethlehem in Free State, and Silverton in Pretoria during the 2022/23 growing season. The experiments were laid out in a 5 × 10 alpha lattice design with two replications. The experimental plot size was a row of 2-meter-long with inter-row and intra-row spacing of 90 cm and 25 cm, respectively. Standard agronomic practices were carried out following sorghum production guidelines in South Africa [17]. During the growing season, the trials were kept weed free and supplemented with irrigation.

**Table 1:** List of sorghum genotypes used in the study

Name	Genotype designation	Source	Seed colour	Country	Name	Genotype designation	Source	Seed colour	Country
05-POTCH-138	50-POTCH-138	ARC-GCI	White	SA	**AS143**	Red Swazi	ACCI	Brown	SA
16MZ	-	-	Brown	-	**AS145**	AWN98	ACCI	Brown	SA
AS106	Landrace	ACCI	Cream	SA	**AS147**	MRS94	ACCI	Red	SA
AS108	P9504B	ACCI	Cream	SA	**AS148**	SDS 3472	ACCI	Brown	SA
AS109	P9511B	ACCI	Cream	SA	**AS152**	01MN1589	ACCI	Brown	SA
AS111	P9539B	ACCI	Cream	SA	**AS194**	Mtentu Imphe	D Vatcha	Brown	-
AS113	TX2737/91BE7414	ACCI	Cream	SA	**AS203**	SA landrace LP 49	J M Donaldson	Brown	SA
AS114	BTx3197	ACCI	Cream	SA	**AS205**	SA landrace LP 51	J M Donaldson	Brown	SA
AS115	BTx631	ACCI	Cream	SA	**AS251**	AS97 OPV	ACCI	Red	SA
AS116	01Aphid207	ACCI	Cream	SA	**AS391**	SS27 OPV	Mtentu	Brown	SA
AS117	01Aphid148	ACCI	Cream	SA	**AS449**	#12 235,926 OC	Ethiopia	Red	Ethiopia
AS121	Kat 369 x EX-1 Chira	ACCI	Brown	SA	**AS560**	IESV 92,028 DL	ICRISAT	Brown	-
AS122	KSV 12	ACCI	Cream	SA	**AS563**	IS 2331	ICRISAT	Brown	-
AS129	KARI Mtama X ICS 3 − 1	ACCI	Cream	SA	**AS72**	KAT-487	UK-SGVT 07–49	Cream	-
AS130	Gambella 1107	ACCI	Cream	SA	**AS74**	ICSV 111	UK-SGVT 07–51	Brown	-
AS131	WK#1025 Sudan	ACCI	Cream	SA	**G50**	TZA 5557	Tanzania	Brown	Tanzania
AS132	Parc 1,260,793	ACCI	Cream	SA	**ICS634**	-	ICRISAT	Brown	-
AS133	Marimanti Co 1110	ACCI	Cream	SA	**ICSV92001**	-	ICRISAT	Brown	-
AS134	P6 NQ#23 Sudan	ACCI	Brown	SA	**LP4403**	LP4403	ARC-GCI	Brown	SA
AS135	Dinkmash	ACCI	Cream	SA	**MAMOLOKWANE**	Mamolokwane	ARC-GCI	White	SA
AS136	FLO (107) x GS 3541	ACCI	Cream	SA	**NW5393**	-	ARC-GCI	Brown	SA
AS137	IESV 92,022 DL	ACCI	Grey	SA	**NW5430**	-	ARC-GCI	Brown	SA
AS138	Mugeta	ACCI	White	SA	**PAN8816**	PAN8816	Pannar	Red	SA
AS140	Kaguru	ACCI	Red	SA	**SS27**	SS27	ARC	Brown	SA
AS141	Kiboko loca	ACCI	Red	SA	**SV07002**	-	ICRISAT	Brown	-

*ARC-GCI* Agricultural Research Council – Grain Crops Institute; *ACCI* African Centre for Crop Improvement; *UKZN* University of KwaZulu-Natal; *ICRISAT* International Crops Research Institute for the Semi-Arid Tropics; *SA* South Africa; *-* = unknown

### Data collection

#### Agronomic traits

Data were collected on the following agronomic traits: days to 50% heading (DTH) measured as the number of days from planting to when 50% of the genotypes in each plot had fully exerted panicles; days to 50% maturity (DTM) measured as the number of days from planting to when 50% of the genotypes in each plot had dried panicles; plant height (PH) was measured from the ground to the tip of the panicle at maturity and expressed in cm. Shoot biomass (SB) was measured as the total mass of the above-ground biomass cut from the base of the plant, excluding the grain (g plant^− 1^). The shoots were oven-dried at 70 °C for 48 h and weighed. Root biomass (RB) was measured as the total root dry matter harvested per genotype per plot (g plant^− 1^). Root samples for each genotype were harvested to a depth of 50 cm. The roots were separated from the soil by hand and washed under running water to remove all soil particles. The roots were oven-dried at 60 °C for 72 h and weighed. Total plant biomass (PB) was the sum of all dry plant material for each genotype, including RB and SB harvested from the test plots (g plant^− 1^). Root-to-shoot biomass ratio (RS) was the ratio of the root and shoot biomass. Grain yield (GY) was the weight of harvested grain at 12.5% moisture content per genotype per plot (g plant^− 1^). Harvest index (HI) was computed as the proportion of grain production to above-ground plant biomass (%).

#### Carbon storage traits

Due to the high cost of sample transportation, preparation, and carbon analysis, among the 50 sorghum genotypes, 25 were selected from the Silverton trials based on their grain yield performance. The 25 lines were subjected to carbon analysis using two replications. Shoot, root, and grain samples were collected to determine shoot carbon content (SCc) (%), root carbon content (RCc) (%), and grain carbon content (GCc) (%), respectively. These samples were oven-dried at 70 °C for 48 h and ground into a fine powder using a ZM 200 ultra centrifugal mill [18], weighing five grams each sample. The samples were submitted to the South African Sugarcane Research Institute (SASRI) in Durban for carbon analysis. The total carbon content of the samples was determined at SASRI by combustion using a LECO TruMac CNS Analyzer. The carbon content of the samples was subsequently converted to shoot carbon stock (SCs) (g plant^− 1^), root carbon stock (RCs) (g plant^− 1^), and grain carbon stock (GCs) (g plant^− 1^). The C stocks in the two parts (SCs and RCs) were summed up to derive total plant carbon stocks (PCs) (g plant^− 1^).

### Data analysis

Data were subjected to analysis of variance using the Statistical Analysis System (SAS) software version 9.4 program with the lattice procedure [19]. A combined analysis of variance was conducted after testing the homogeneity of variance using Levene’s test [20]. Variance components for agronomic and carbon storage traits were estimated based on combined and single environment analyses, respectively.

#### Estimation of genotypic and phenotypic variance

Genotypic and phenotypic variance for agronomic traits were calculated from the results of combined analysis of variance according to Rahimi and Hernandez [21]:

$$\:{\sigma\:}_{e}^{2}=\:MSe$$

$$\>\sigma _{ge}^2\> = \>{\rm{(}}{{MSge - MSe} \over r}{\rm{)}}$$

$$\sigma \>_g^2 = \>{\rm{(}}{{MSg - MSge} \over {r{\rm{*}}\>e}}{\rm{)}}$$

$$\sigma \>_p^2 = \>\sigma \>_g^2\> + \>\sigma \>_{ge}^2\> + \>\sigma \>_e^2$$

Where; $\sigma \>_{\rm{e}}^2$ is the environmental variance of a particular trait; $\sigma \>_{{\rm{ge}}}^2$ is the genotype x environment interaction variance of a particular trait; $\sigma \>_{\rm{g}}^2$ is the genotypic variance of a particular trait; $\sigma \>_{\rm{p}}^2$ is the phenotypic variance of a particular trait; MS_e_ is the mean square of the environment; MSg_e_ is the mean square of genotype x environment; MS_g_ is the mean square of genotype; r = number of replications; and e = number of environments.

Genotypic and phenotypic variance for carbon storage traits were calculated from the results of a single environment analysis of variance, as suggested by Burton and Devane [22].

$$\sigma _\varepsilon ^2 = MS\varepsilon$$

$$\sigma \>_g^2 = {{MSg - MS\varepsilon \>} \over {r\>}}$$

$$\sigma \>_p^2 = \sigma \>_g^2\> + \sigma \>_{\varepsilon \>}^2$$

Where $\:MS\varepsilon\:$ is the mean square error and $\sigma \>_{\varepsilon \>}^2$ is the error variance component.

#### Estimation of coefficient of variability

Genotypic coefficient of variation (GCV) and phenotypic coefficient of variation (PCV) were computed according to Burton and Devane [22]:

$$GCV\> = \left( {{{\sigma {\>_g}} \over {\bar x}}} \right)\>*\>100$$

$$\>PCV\> = \>\left( {{{\sigma {\>_p}} \over {\bar x}}} \right)*\>100$$

Where $\sigma {\>_{\rm{g}}}$ is the genotypic standard deviation of a particular trait; ${\sigma _{\rm{p}}}$ is the phenotypic standard deviation of a particular trait; and x̅ is the mean performance of a particular trait.

#### Heritability and genetic advance

The broad-sense heritability of a given trait at a single environment was calculated according to Alvarado et al. [23]:

$$H2\> = {{\sigma \>_g^2} \over {\sigma \>_g^2\> + \>\sigma \>_{\varepsilon \>}^2\>/r\>}}$$

Where $\sigma \>_{\rm{g}}^2$ is the genotypic variance; $\sigma \>_{\varepsilon \>}^2$ is the error variance component; and r is the number of replications, whereas regarding combined analyses, broad-sense heritability was calculated as:

$$H2\> = {{\sigma \>_g^2} \over {\sigma \>_g^2 + \>\sigma \>_{ge}^2/e\> + \>\sigma \>_{\varepsilon \>}^2\>/\>\left( {r\>{\rm{*}}\>e} \right)\>\>\>}}$$

Where $\sigma \>_{{\rm{ge}}}^2$ is the genotype x environment interaction and e is the number of environments.

Genetic advance (GA) and the genetic advance as a percent of the mean (GAM) were computed using the following formulae [24]:

$$GA\> = KH^2\sigma \>_{p\>}$$

$$GAM\> = \left( {{{GA} \over {\bar x}}\>} \right)*\>100$$

Where K is 2.06 at 5% selection intensity.

#### Correlation and path coefficient analyses

The phenotypic and genotypic correlation coefficients for agronomic and carbon storage traits were computed based on the procedure of Dabholkar [25].

Genotypic correlation coefficients were estimated as follows:

$$rg\; = \;COVg\;(xy)/\;\sigma g\;(x) * \;\sigma g\;(y)$$

While phenotypic correlation coefficients were estimated as follows:

$$\:rph\:=COVph\:\left(xy\right)/\:\sigma\:ph\:\left(x\right)\:*\:\sigma\:ph\:\left(y\right)$$

Where, COV_g_ (xy) and COV_ph_ (xy) are the genotypic and phenotypic covariances of two variables (x and y), respectively; σ_g_ (x) and σ_g_ (y) are the genotypic standard deviations for variables, x and y, respectively; and σ_ph_ (x) and σ_ph_ (y) are the phenotypic standard deviations of variables, x and y, respectively.

The significance of genotypic correlation coefficients were ascertained following Robertson [26]. The significance of phenotypic correlation coefficients was tested using procedures proposed by Singh and Chaudhary [27]. Path coefficient analysis involved estimating the genotypic and phenotypic direct and indirect effects of independent traits on the dependent trait (grain yield) using methods reported by Dewey and Lu [28].

$$\:rij\:=\:Pij\hspace{0.17em}+\hspace{0.17em}rik\:Pkj$$

Where, r_ij_ is the association between the independent variable (i) and dependent variable (j) as measured by correlation coefficient; P_ij_ is the component of the direct effect of the independent variable (i) on the dependent variable (j) as measured by path coefficient; and ∑r_ik_ P_kj_ is the summation of components of indirect effects of a given independent variable (i) on a given dependent variable (j) via all other independent variables. The residual factor (P^2^R) was estimated as described in Dewey and Lu [28]:

$$\:1=\:P2R\:+\:\sum\:Pij\:rij\:$$

## Results

### Combined analysis of variance for agronomic traits

A combined analysis of variance showing the mean square values and significant tests is presented in Table 2. Significant (*p* < 0.05) differences were calculated among genotypes for all the assessed agronomic traits except DTM while significant genotype by location interactions were recorded for PH and PB.

**Table 2:** Combined analysis of variance showing mean squares and significance tests for agronomic traits of 50 sorghum genotypes across three locations in South Africa

Source of variation	DF	DTH	DTM	PH	PB	SB	RB	RS	GY	HI
Location	2	91.83	349.41	20546.17***	20.14	18.49	22.83**	0.004	13.70**	50.34
Replication	3	479.85	519.62**	28833.24***	9.61	7.93	0.37	0.01	7.43*	18.37
Block	24	167.77	111.44	2757.55	25.17	11.53	3.31	0.005	4.42*	21.91
Genotype	49	307.42*	130.78	3194.9**	349.57***	151.72***	129.22***	0.26***	181.77***	1031.47***
Genotype x location	98	207.06	111.63	2257.43**	18.36*	14.44	4.5	0.004	2.57	15.34
Error	123	195.51	135.71	1826.3	15.99	14.52	5.06	0.004	2.73	17.83

*, ** and *** denote significance at *p* < 0.05, *p* < 0.01, and *p* < 0.001, respectively; *DF* degrees of freedom; *DTH* days to 50% heading; *DTM* days to 50% maturity; *PH* plant height (cm); *PB* total plant biomass (g plant^− 1^); *SB* shoot biomass (g plant^− 1^); *RB* root biomass (g plant^− 1^); *RS* root to shoot biomass ratio; *GY* grain yield (g plant^− 1^); *HI* harvest index (%)

### Analysis of variance for carbon storage traits

The analysis of variance revealed significant differences (*p* < 0.05) among genotypes for GCc, PCs, SCs, RCs, RCs/SCs, and GCs (Table 3).

**Table 3:** Analysis of variance showing mean squares and significance tests for carbon storage of the 25 selected sorghum genotypes at Silverton during 2022 growing season

Source of variation	DF	SCc	RCc	GCc	PCs	SCs	RCs	RCs/SCs	GCs
Replication	1	1.24	5.21	0.03	1.34	2.85	0.29	0.004	0.86
Block	8	0.28	7.45	0.01	10.88	13.41	0.38	0.05*	0.21
Genotype	24	0.65	12.77	0.49***	29.02**	20.13*	5.47***	0.13***	8.44***
Error	16	0.32	8.03	0.01	8.76	7.61	0.42	0.02	0.34

*, ** and *** denote significance at *p* < 0.05, *p* < 0.01, and *p* < 0.001, respectively; *DF* degrees of freedom; *SCc* shoot carbon content (%); *RCc* root carbon content (%); *GCc* grain carbon content (%); *PCs* total plant carbon stocks (g plant^− 1^), *SCs* shoot carbon stock (g plant^− 1^); *RCs* root carbon stock (g plant^− 1^); *RCs/SCs* root to shoot carbon stock ratio; *GCs* grain carbon stock (g plant^− 1^)

### Genotype response for grain yield and component traits and carbon storage

The mean grain yield of the 50 genotypes observed for the genotypes was 11.9 g plant^− 1^ (Table 4). The following high yielding genotypes were selected: AS115, AS251, AS134, AS145, and AS130 with mean yields of 25.08 g plant^− 1^, 21.83 g plant^− 1^, 21.42 g plant^− 1^, 19.43 g plant^− 1^, and 18.50 g plant^− 1^, respectively. DTM ranged from 128 to 151 days. The study selected early maturing genotypes, such as AS115 and AS230 with 130 and 131 maturation days, respectively. Genotypes AS115 and AS111 had tall plant stature with a mean PH of 182.5 cm and 185.67 cm, respectively. The genotypes that had the highest SB and RB were among the top performing genotypes. Genotype SS27 had the highest PB and SB with mean values of 38.65 g plant^− 1^ and 24.87 g plant^− 1^, in that order. The highest RB was recorded for genotypes AS134 and AS130, with mean values of 15.16 g plant^− 1^ and 14.27 g plant^− 1^, respectively. Genotype AS130 had the highest root-to-shoot biomass ratio (1.26) compared to all the genotypes.

**Table 4:** Mean performances among the ten best and five bottom sorghum genotypes ranked based on grain yield and genetic parameters for agronomic traits in 50 genotypes evaluated at three locations during the 2022/23 growing seasons in South Africa

Genotype	DTH	DTM	PH	PB	SB	RB	RS	GY	HI
**Top ten genotypes**									
AS115	88	130	182.50	21.39	12.54	8.85	0.71	25.08	66.66
AS251	85	142	131.39	27.60	14.63	12.97	0.89	21.83	59.88
AS134	78	135	162.72	35.52	20.37	15.16	0.74	21.42	51.26
AS145	86	141	157.67	32.20	21.95	10.25	0.47	19.43	46.95
AS130	77	145	163.33	25.56	11.29	14.27	1.26	18.50	62.09
SS27	79	142	135.92	38.65	24.87	13.77	0.55	17.58	41.41
AS138	78	140	146.17	28.54	15.03	13.51	0.90	17.45	53.73
AS132	94	133	171.06	25.90	15.61	10.29	0.66	16.89	51.98
AS563	84	135	173.94	33.02	21.31	11.71	0.55	16.83	44.12
AS203	82	131	174.50	38.19	23.45	14.74	0.63	16.63	41.49
**Bottom five genotypes**									
AS147	80	138	145.22	16.20	7.51	8.69	1.16	4.63	38.13
AS116	77	138	142.11	21.52	14.89	6.63	0.45	4.35	22.62
PAN8816	75	140	107.06	33.58	17.47	16.12	0.92	3.75	17.68
AS129	84	143	132.28	26.89	17.41	9.49	0.54	3.28	15.87
AS111	84	144	185.67	23.22	13.97	9.25	0.66	2.53	15.34
Mean	81.23	138.7	155.8	31.74	17.44	11.25	0.59	10.69	30.39
SD	14.37	11.17	57.94	8.26	7.375	4.57	0.25	6.31	13.92
SE	1.52	1.18	6.11	0.87	0.78	0.48	0.03	0.66	1.47
**Overall statistics of the 50 genotypes**									
Mean	79.52	138.01	156.01	27.94	16.31	11.62	0.8	11.9	41.47
$\:{\sigma\:}_{g}^{2}$	16.73	3.19	156.25	55.2	22.88	20.79	0.04	29.87	169.36
$\:{\sigma\:}_{p}^{2}$	166.17	233.71	11558.05	74.45	39.35	34.45	0.11	38	202.2
$\:{\sigma\:}_{e}^{2}$	91.83	349.41	20546.17	20.14	18.49	22.83	0.05	13.7	50.34
$\:{\sigma\:}_{ge}^{2}$	57.62	-118.89	-9144.37	-0.89	-2.03	-9.17	0.02	-5.57	-17.5
GCV (%)	5.14	1.29	8.01	26.59	29.33	39.24	25.82	45.92	31.38
PCV (%)	16.21	11.08	68.91	30.88	38.46	50.51	41.96	51.8	34.29
H^2^%	10.07	1.37	1.35	74.14	58.15	60.34	37.87	78.59	83.76
GA (%)	2.67	0.43	2.99	13.18	7.51	7.3	0.41	9.98	24.53
GAM	3.36	0.31	1.92	47.17	46.07	62.78	50.86	83.87	59.16

*SD* standard deviation; *SE* standard error; *DTH* days to 50% heading; *DTM* days to 50% maturity; *PH* plant height (cm); *PB* total plant biomass (g plant^− 1^); *SB* shoot biomass (g plant^− 1^); *RB* root biomass (g plant^− 1^); *RS* root to shoot biomass ratio; *GY* grain yield (g plant^− 1^); *HI* harvest index (%); *σ*^*2*^_*p*_ phenotypic variance; *σ*^*2*^_*g*_ genotypic variance; *σ*^*2*^_*e*_ environmental variance; *σ*^*2*^_*ge*_ genotype by environment interaction variance; *PCV* phenotypic coefficient of variation; *GCV* genotypic coefficient of variation; *H*^*2*^ heritability; *GA* genetic advance; *GAM* genetic advance as a percentage of the mean

The mean total plant carbon stock for the test genotypes was 12.95 g plant^− 1^ (Table 5). The highest PCs were recorded for SS27, AS122, AS134, and AS203 with values of 24.64 g plant^− 1^, 18 g plant^− 1^, 16.48 g plant^− 1^, and 15.55 g plant^− 1^, respectively. The four genotypes were also among the highest grain yield producers. The genotypes that allocated the highest carbon in shoots were SS27, AS122, ICSV92001, and AS563 at19.04 g plant^− 1^, 10.42 g plant^− 1^, 10.34 g plant^− 1^, 10.33 g plant^− 1^. Almost all the genotypes stored more carbon in the shoots than in the roots. The genotype that stored more carbon in the roots compared to shoots was AS108, with the RCs value of 8.87 g plant^− 1^ and the highest RCs/SCs of 1.56.

**Table 5:** Mean performances among the ten best and five bottom sorghum genotypes ranked based on their total plant carbon stock and genetic parameters for carbon storage traits in 25 selected sorghum genotypes at Silverton during the 2022/23 growing season

Genotype	SCc	RCc	GCc	PCs	SCs	RCs	RCs/SCs	GCs
**Top ten genotypes**								
SS27	45.48	39.92	43.27	24.64	19.04	5.61	0.29	7.61
AS122	43.62	40.39	43.43	18	10.42	7.58	0.73	3.69
AS134	44.41	39.34	43.25	16.48	9.34	7.13	0.76	11.38
AS203	44.56	43.41	43.47	15.55	9.15	6.4	0.7	7.23
AS563	44.7	40.26	44.49	14.99	10.33	4.66	0.45	5.91
AS251	43.51	43.26	42.89	14.97	8.48	6.49	0.76	4.57
16MZ	43.2	44.41	43.24	14.92	9.83	5.09	0.52	5.24
AS108	43.74	44.79	43.96	14.55	5.68	8.87	1.56	3.92
ICSV92001	44.55	39.94	44.65	14.33	10.34	3.99	0.39	3.35
LP4403	43.73	43.76	45.31	13.89	8.38	5.51	0.66	3.35
**Bottom five genotypes**								
AS138	44.87	40.72	43.36	8.52	5.35	3.17	0.59	8.04
AS143	44.68	43.67	44.24	8.16	5.41	2.76	0.51	3.87
NW5393	45.09	39.4	44.07	7.96	5.2	2.75	0.53	5.73
AS116	43.78	40.12	43.34	7.69	6.33	1.37	0.22	1.78
AS115	43.44	45.34	43.56	7.52	3.25	4.27	1.31	12.92
Mean	44.25	41.86	43.78	12.95	8.32	4.63	0.62	5.23
SD	0.81	2.74	0.65	5.56	4.57	2.31	0.35	2.21
SE	0.15	0.5	0.12	1.02	0.83	0.42	0.06	0.40
**Overall statistics of the 25 genotypes**								
Mean	44.18	41.1	43.65	12.65	7.98	4.67	0.65	5.84
$\:{\sigma\:}_{g}^{2}$	0.17	2.37	0.24	10.13	6.26	2.53	0.06	4.05
$\:{\sigma\:}_{p}^{2}$	0.49	10.4	0.25	18.89	13.87	2.95	0.08	4.39
GCV (%)	0.92	3.75	1.12	24.88	30.51	34.62	38.45	40.25
PCV (%)	1.58	7.85	1.15	33.98	45.42	37.39	44.9	41.9
H^2^ (%)	34.02	22.79	96	53.63	45.13	85.74	73.33	92.26
GA (%)	0.49	1.51	0.99	4.8	3.46	3.03	0.41	3.98
GAM (%)	1.1	3.68	2.27	37.54	42.23	66.03	67.82	79.64

*SD* standard deviation; *SE* standard error; *SCc* shoot carbon content (%); *RCc* root carbon content (%); *GCc* grain carbon content (%); *PCs* total plant carbon stocks (g plant^− 1^), *SCs* shoot carbon stock (g plant^− 1^); *RCs* root carbon stock (g plant^− 1^); *RCs/SCs* root to shoot carbon stock ratio; *GCs* grain carbon stock (g plant^− 1^); *σ*^*2*^_*p*_ phenotypic variance; *σ*^*2*^_*g*_ genotypic variance; *PCV* phenotypic coefficient of variation; *GCV* genotypic coefficient of variation; *H*^*2*^ heritability; *GA* genetic advance; *GAM* genetic advance as a percentage of the mean

### Variance components, heritability, and genetic advance for agronomic traits

Phenotypic coefficient of variation (PCV), genotypic coefficient of variation (GCV), heritability (H^2^), genetic advance (GA), and genetic advance as a percentage of the mean (GAM) for agronomic traits are presented in Table 4. PB varied from 14.07 to 43.75 g plant^− 1^, and GY ranged from 2.53 to 25.08 g plant^− 1^. HI and PH had the highest genotypic variance of 169.36 and 156.25, respectively. The phenotypic variance was the highest for PH (1158.05), followed by DTM (233.71), and HI (202.2). The highest environmental variance was recorded for PH (20546.17) and DTM (349.41). The trait that exhibited the highest genotype by location interaction variance was DTH, with a value of 57.62, while PH exhibited the lowest genotype by environment interaction of -9144.37. The highest GCV values were recorded for GY (45.92%), RB (39.24%), and HI (31.38%). The highest PCV values were recorded for PH (68.91%), followed by GY (51.8%), RB (50.51%), and RS (41.96%).

The highest heritability values were recorded for HI, GY, PB, and RB at 83.76%, 78.59%, 74.14%, and 60.34%, respectively. Moderate heritability values were computed for shoot biomass (58.15%) and RS (37.87%). The highest genetic advance values were recorded for HI (24.53 g plant^− 1^), followed by PB (13.18 g plant^− 1^), and GY (9.98 g plant^− 1^). The highest GAM values were computed for GY (83.87%) and RB (62.78%). Moderate GAM values were recorded for HI, RS, PB, and SB with values of 59.16%, 50.86%, 47.17%, and 46.07%, respectively. The lowest GAM was recorded for DTH (3.36%), DTM (0.31%), and PH (1.92%).

### Variance components, heritability, and genetic advance for carbon storage traits

PCs, SCs and RCs values ranged from 7.52 to 24.64 g plant^− 1^, 3.25 to 19.04 g plant^− 1^, and 1.37 to 8.87 g plant^− 1^, respectively (Table 5). The highest genotypic variance was recorded for PCs, SCs, and GCs, with values of 10.13, 6.26, and 4.05, respectively. The same trend was observed for phenotypic variance. Higher GCV and PCV values were observed for PCs (24.88 and 33.98%), SCs (30.51 and 45.42%), RCs (34.62 and 37.39%), RCs/SCs (38.45 and 44.90%), and GCs (40.25 and 41.90%) for both GCV and PCV, respectively. The SCc values had GCV of 1.58 and PCV of 34.02%, RCc (3.75 and 7.85%), and GCc (1.12 and 1.15%), respectively.

High heritability values were recorded for GCc, GCs, RCs, and RCs/SCs with 96%, 92.26%, 85.74%, and 73.33%, respectively (Table 5). PCs and SCs had moderate heritability estimates of 53.63% and 45.13%, respectively. Low heritability (< 50%) estimates were computed for SCc and RCc with 34.02%, and 22.79%, respectively.

The highest GA estimates were calculated for PCs (4.8 g plant^− 1^), followed by GCs (3.98 g plant^− 1^), SCs (3.46 g plant^− 1^), and RCs (3.03 g plant^− 1^) (Table 5). SCc, RCc, GCc, and RCs/SCs had very low GA estimates with values of 0.49 g plant^− 1^, 1.51 g plant^− 1^, 0.99 g plant^− 1^, and 0.41 g plant^− 1^, respectively. The GAM estimates were the highest for GCs (79.64%), RCs/SCs (67.82%), and RCs (66.03%). PCs and SCs had GAM estimates of 37.54% and 42.23%, respectively. Very low GAM estimates were recorded for SCc, RCc, and GCc, with values of 1.1%, 3.68%, and 2.27%, respectively.

### Correlations among agronomic traits

Estimates of genotypic and phenotypic correlation coefficients between each pair of agronomic traits are presented in Table 6. Grain yield had positive correlations with DTH, PH, and PB, while negative correlations with DTM, SB, and RS were observed using genotypic and phenotypic correlations. The phenotypic correlation of DTH and DTM was negative compared to the genotypic correlation, which was positive. Plant height had a positive correlation with DTH but had a negative correlation with DTM using genotypic and phenotypic correlations. Plant height also had a positive and significant correlation with SB at the phenotypic level. Total plant biomass exhibited a positive and significant genotypic and phenotypic correlations with SB, RB, RS, and GY and a negatively significant correlation with HI at both phenotypic and genotypic levels. Root biomass had a positive and highly significant correlation with PB using genotypic and phenotypic correlations. It had a positive and significant correlation with RS using genotypic and phenotypic correlations. Harvest index exhibited a positive and significant correlation with RS and GY; and had a negative and significant correlation with PB and SB using genotypic and phenotypic correlations.

**Table 6:** Genotypic (above diagonal) and phenotypic (below diagonal) correlation coefficients among agronomic traits in 50 sorghum genotypes

Trait	DTH	DTM	PH	PB	SB	RB	RS	GY	HI
DTH	**1**	0.052	0.232***	0.033	0.05061	-0.007	-0.049	0.127*	0.052
DTM	-0.12	**1**	-0.161**	-0.049	-0.02821	-0.046	-0.042	-0.054	-0.051
PH	0.543***	-0.435**	**1**	0.079	0.117*	-0.01	-0.08	0.096*????	0.026
PB	0.043	-0.152	0.182	**1**	0.796***	0.685***	0.013	0.01**	-0.424***
SB	0.074	-0.036	0.174	0.771***	**1**	0.105	-0.527***	-0.03	-0.586***
RB	-0.014	-0.199	0.095	0.720***	0.113	**1**	0.657***	0.053***	0.008
RS	-0.078	-0.196	-0.073	0.048	-0.548***	0.672***	**1**	-0.021	0.383***
GY	0.306*	-0.08	0.267**	0.015***	-0.033	0.06*	-0.02	**1**	0.764***
HI	0.127	-0.118	0.114	-0.388**	-0.579***	0.026	0.37***	0.786***	**1**

*, ** and *** denote significance at *P* < 0.05, *P* < 0.01, and *P* < 0.001, respectively; *DTH* days to 50% heading; *DTM* days to 50% maturity; *PH* plant height; *PB* total plant biomass; *SB* shoot biomass; *RB* root biomass; *RS* root to shoot biomass ratio; *GY* grain yield; *HI* harvest index

### Correlations among carbon storage variables

Genotypic and phenotypic correlation coefficients between carbon storage traits are shown in Table 7. The total plant carbon stock had a positive genotypic and phenotypic correlation with SCc; and exhibited positive and significant correlations with SCs, RCs, and GCs at both genotypic and phenotypic levels. Shoot carbon content had a negative and significant genotypic and phenotypic correlation with RCc, while exhibiting positive correlations with PCs, SCs, and GCs at both genotypic and phenotypic levels. Root carbon content exhibited a positive and significant genotypic and phenotypic correlation with RCs/SCs. Grain carbon stock had a positive and significant correlation with all the assessed traits except for RCc for both genotypic and phenotypic levels.

**Table 7:** Genotypic (above diagonal) and phenotypic (below diagonal) correlation coefficients among carbon storage traits in 25 selected sorghum genotypes

Traits	SCc	RCc	GCc	PCs	SCs	RCs	RSC/SCs	GCs
SCc	**1**	-0.303*	0.112	0.094**	0.154**	-0.077	-0.105	0.113*
RCc	-0.518**	**1**	0.102	-0.129	-0.257	0.196	0.354**	-0.125
GCc	0.09	0.14	**1**	-0.077	-0.155	0.118	0.187	0.057***
PCs	0.185**	-0.109	-0.086	**1**	0.910***	0.593***	0.021	0.077**
SCs	0.309*	-0.273	-0.186	0.894***	**1**	0.207	-0.364**	0.03**
RCs	-0.106	0.211	0.117	0.687***	0.289	**1**	0.758***	0.122*
RCs/SCs	-0.175	0.371	0.194	0.138	-0.295	0.773***	**1**	0.057*
GCs	0.194**	-0.089	0.056***	0.086*	0.032**	0.132*	0.077*	**1**

*, ** and *** denote significance at *P* < 0.05, *P* < 0.01, and *P* < 0.001, respectively; *SCc* shoot carbon content; *RCc* root carbon content; *GCc* grain carbon content; *PCs* total plant carbon stocks, *SCs* shoot carbon stock; *RCs* root carbon stock; *RCs/SCs* root to shoot carbon stock ratio; *GCs* grain carbon stock

### Path coefficient analysis for agronomic traits

Positive and high direct effects on grain yield were recorded when using phenotypic correlations by HI (1.159), SB (0.607), RB (0.546), DTH (0.033), and DTM (0.003), while negative and high direct effects on grain yield were observed by plant height (-0.024), PB (-0.347), and RS (-0.5) (Table 9). The positive and high correlation coefficients of HI and DTH with grain yield were due to the positive indirect effects of DTH (0.002), PB (0.147), and RB (0.005) through HI on grain yield. Similarly, DTH and RB had strong positive correlations and positive direct effects on grain yield, indicating they are more significantly related to grain yield at phenotypic level. Total plant biomass (0.374) and RS (0.358) had the greatest positive indirect effects on grain yield through RB. Shoot biomass (-0.277)) and root biomass (-0.238) had the highest negative indirect effects on grain yield through PB. Root biomass (-0.328), HI (-0.192), and PB (-0.007) exhibited negative and high indirect effects on grain yield through RS. Harvest index (0.445) exerted the positive and highest indirect effect on grain yield through RS. The residual effect for the phenotypic path coefficient analysis was 0.095.

The genotypic direct and indirect effects of different agronomic traits on grain yield are presented in Table 9. Positive and high direct effects on grain yield were recorded by HI (1.136), SB (0.393), RB (0.31), DTH (0.131), and DTM (0.04), while negative and high direct effect was recorded by PH (-0.037), PB (-0.043), and RS (-0.418). DTH had positive and indirect effects of 0.071 and 0.017 on grain yield, which can be selected through PH and HI. Shoot biomass (0.303) and root biomass (0.224) had the highest positive indirect effects on grain yield through PB. Negative indirect effects on grain yield through RB were observed for DTH (-0.004) and DTM (-0.062). Root-to-shoot biomass ratio had negative indirect effects on grain yield through PB (-0.02), RB (-0.281), and HI (-0.155). The residual effect for genotypic path coefficient analysis was 0.066.

## Discussion

### Estimation of genetic parameters for the agronomic and carbon storage traits

The results suggest that the test genotypes displayed adequate genetic variation for selection for enhanced biomass production, grain yield, and carbon allocation to roots and shoots. In agreement with the present results, Abraha et al. [29] reported high PCV and GCV for sorghum grain yield, biomass, and carbon storage traits. The highest PCV and GCV values were recorded for GY, followed by RB, RCs/SCs, and RCs supporting the agronomic traits (Tables 4 and 5). Narrow differences between PCV and GCV values were recorded for SB, RB, GY, HI, SCc, GCc, and GCs, indicating the minimal impact of the test locations on genotype selection [30]. These results are consistent with those of Haussmann et al. [31] and Ayele [32], who found the same trend for sorghum grain yield and those of Kishore and Singh [33] for biomass yield. Ayana et al. [34] and Munzbergova et al. [35] reported that genotypic variation is influenced by rainfall, temperature, and growing site gradients. McGuigan and Sgro [36] argued that phenotypic expression can reveal genetic heterogeneity and is subject to environmental influences.

Heritability refers to the proportion of phenotypic variance attributed to genetic variance. High to moderate broad sense heritability values were computed for PB, GY, HI, RB, GCc, GCs, RCs, RCs/SCs, SB, RS, and PCs (Tables 4 and 5). The results suggest that these traits will have higher response to selection, being less influenced by environmental effects. Related results were observed in sorghum for grain yield and biomass [29]. Days to 50% heading, days to 50% maturity, and plant height, shoot carbon content, and root carbon content showed low heritability. Phenotypic selection for low heritable traits can be less effective, needing indirect selection via traits with higher heritability or the use of complementary high throughput molecular markers. These results conform with the findings of Gebregergs and Mekbib [37] who reported low heritability in sorghum for days to 50% maturity. Low heritability traits can be selected using molecular markers linked to quantitative trait loci (QTLs) for the target traits, allowing individuals to be graded based on their genetic makeup rather than their phenotypic characteristics [38]. Previous findings have indicated a complex inheritance pattern for days to 50% heading and days to 50% maturity, conditioned by dominant and recessive genes [39]. A putative dwarfing locus was found through genome-wide association studies (GWAS) [40], and the locus has been linked to higher total plant biomass and root biomass [41]. These findings indicate that the locus may have a pleiotropic effect on carbon storage and partitioning [42]. Heritability value helps determine the success of selection via phenotypic traits [43]. The high heritability estimate observed for RCs/SCs suggests the effectiveness of selection using the root-to-shoot ratio for enhancing carbon allocation and genetic gain in roots and shoots.

Characters with high heritability can easily be successfully selected, resulting in quick genetic progress. However, it has been accentuated that heritability alone has less practical importance without genetic advancement due to the reliance on genetic variability and the possibility of unexpected environmental interactions altering characteristic expression over time [44]. Genetic advance (GA) refers to the degree of gain obtained in a trait under a particular selection pressure. High GA was recorded for PB, GY, and HI in this study (Table 4). Similar results were reported by Mofokeng et al. [12] in sorghum for grain yield and thousand seed weight. The highest heritability and genetic advance as a percentage of the mean (GAM) was recorded for RB, followed by GY, HI, RCs, RCs/SCs, and GCs. High GAM is associated with effective selection of sorghum genotypes with high yield and carbon storage. High heritability and genetic advance values indicate the presence of additive gene action, which are highly heritable and suggesting that crop improvement can be achieved by selecting such traits [45–48]. Estimates of heritability and genetic advance are more reliable and informative [49, 50]. Priority should be given to traits that displayed high heritability and genetic advances to develop accurate selection indices for developing sorghum genotypes with high grain yield and carbon sequestration potential.

### Correlations among agronomic and carbon storage traits

Grain yield is a complex polygenic trait influenced by various yield component traits. Assessing the association between these traits and their correlation with grain yield helps establish effective selection strategies [51]. This study examined the trend and magnitude of relationships between sorghum agronomic and carbon storage traits to identify grain yield and carbon storage contributing traits. The results indicated a strong association among the assessed traits (Tables 6 and 7). Traits such as DTH, PH, PB, and HI had a positive and significant correlation with grain yield (Table 6). These are proxy traits to be used for yield improvement in sorghum breeding programs. Plant height had a positive and significant correlation with DTH but exhibited a significant and negative correlation with DTM using genotypic and phenotypic correlations (Table 6). PH positively correlated with SB when using both genotypic and phenotypic correlations. Hence, improving PH, PB, and SB will significantly increase grain yield and carbon storage in sorghum genotypes. These results concord with the findings of Amare et al. [52], who reported a positive and significant association between HI and grain yield in sorghum when genotypic and phenotypic correlations were assessed. Traits such as SB, RB, RS, and GY exhibited a positive and significant genotypic and phenotypic correlations with PB, signifying their importance in improving this trait. Similarly, positive and significant correlations were reported among grain yield, spikelet per spike, and fresh biomass in wheat genotypes [53]. Therefore, increasing SB and RB can improve vegetative growth, resulting in increased plant biomass production and carbon storage, as increased plant biomass results in increased plant leaf area available for photosynthesis and thus increasing production of photo-assimilates needed for grain filling [54]. Grain yield had positive and significant correlations with all measured traits except for DTM, SB, and RS at both genotypic and phenotypic levels (Table 6). These findings revealed that an increase in the performance of all these traits could increase grain yield and carbon sequestration potential. George-Jaeggli et al. [55] reported that reduced shoot biomass can affect grain yield by decreasing grain size, and variations in carbon allocation in the shoot biomass contribute to trade-offs between structural and non-structural carbohydrate content [56, 57]. Carbon allocation is also influenced by environmental factors, such as drought stress, which causes plants to transition between vegetative and reproductive periods [58]. There is a need to analyse carbon sinks in grain or biomass yields to understand the associations and trade-offs among traits related to carbon allocation. This involves macro-scale phenotyping of traits like grain yield, above-ground biomass and plant height and micro-scale evaluation of compositional traits using techniques such as near-infrared spectroscopy [59].

Root-to-shoot biomass ratio had a positive and highly significant correlation with RB but exhibited a negative and significant correlation with SB at both genotypic and phenotypic levels (Table 6). In agreement with the present results, Mathew et al. [60] and Shamuyarira et al. [61] reported that RS exhibited negative correlations with all agronomic traits except for RB in wheat. According to optimal partitioning theory, plants allocate resources between shoots and roots to promote plant growth [62]. In this regard, plants may exhibit a specific root-to-shoot ratio, balancing resource limitations and implying genetic plasticity or responsiveness [63]. Changes in the root-to-shoot ratio occur during plant growth and development and in response to resource limitations both above and below ground. Thus, it is essential to carefully manage and account for plant size and ontology to draw accurate conclusions about root-to-shoot allocation [62]. Root biomass had a positive and highly significant correlation with PB using the phenotypic analysis and a positive and highly significant correlation with RS and GY at the genotypic level, indicating the importance of root system size in crop biomass and grain yield production (Table 6). The results are consistent with previous studies in sorghum genotypes [64–66]. Ehdaie et al. [67] stated that a larger root biomass may benefit plants in water-limited conditions. Wasson et al. [68] indicated that bigger root systems limit the amount of assimilates available for grain production. Some studies have reported that a carbon imbalance between root and shoot biomass results in lower sorghum yields [69, 70]. Increasing RS alone in pursuit of larger carbon inputs may negatively affect grain production. Therefore, considering the balance between root and shoot biomass is essential to optimize biomass production, grain yield, and carbon sequestration potential. There is a need to resolve the negative relationship between below-ground root characteristics and grain yield. This is feasible if contrasting genotypes with below-ground (e.g. deep and large roots) and above-ground (e.g. tall stature and increased leaf area) characteristics are inter-crossed, followed by a selection of best-recombined individuals [71].

### Path coefficient analysis of agronomic traits

The present findings found that GY had positive and significant correlations with DTH, PB, RB, and HI using path analysis through phenotypic and genotypic correlations (Tables 8 and 9). This revealed the actual contribution of agronomic traits to improving grain yield. The study found it crucial to partition the correlation between grain yield and component traits into direct and indirect effects. This allowed to dissect the intricate relationships between grain yield and its component traits to understand whether the influences reflect directly on yield or take some other pathway that ultimately impacts yield and use the most yield-contributing traits for direct and indirect selection. In the current study, positive and high direct effects were exerted on grain yield by DTH, DTM, SB, RB, and HI while negative and high direct effect was exerted by PH, PB, and RS using both phenotypic and genotypic pathways (Tables 8 and 9). The slightly high and positive correlation coefficient between grain yield and DTH was due to its high indirect effects through PH and HI on grain yield (Table 9). These results are consistent with Shivaprasad et al. [72], who reported a slightly high positive correlation coefficient between grain yield and days to 50% flowering among mutants of sorghum. The residual factors of the genotypic (0.095) and (0.066) phenotypic path coefficient analysis indicate that traits included in the path analysis explained 90.5% and 93.4% of the total variability in grain yield at genotypic and phenotypic levels, respectively. This suggests the repeatability of selection in the present study.

**Table 8:** Genotypic direct effect (bold-faced diagonal values) and indirect effect (off-diagonal) of eight agronomic traits on grain yield of 50 sorghum genotypes

Traits	DTH	DTM	PH	PB	SB	RB	RS	HI	rg
DTH	**0.0327**	0.0002	-0.0055	-0.0114	0.0307	-0.0039	0.0243	0.0602	0.1273
DTM	0.0017	**0.0030**	0.0038	0.0169	-0.0171	-0.0251	0.0210	-0.0587	-0.0545
PH	0.0076	-0.0005	**-0.0239**	-0.0276	0.0707	-0.0055	0.0450	0.0299	0.0958
PB	0.0011	-0.0001	-0.0019	**-0.3474**	0.4830	0.3738	-0.0066	-0.4915	0.0102
SB	0.0017	-0.0001	-0.0028	-0.2767	**0.6065**	0.0572	0.2635	-0.6790	-0.0297
RB	-0.0002	-0.0001	0.0002	-0.2380	0.0636	**0.5456**	-0.3282	0.0098	0.0526
RS	-0.0016	-0.0001	0.0022	-0.0046	-0.3197	0.3583	**-0.4999**	0.4449	-0.0206
HI	0.0017	-0.0002	-0.0006	0.1473	-0.3554	0.0046	-0.1919	**1.1589**	0.7645

*DTH* days to 50% heading; *DTM* days to 50% maturity; *PH* plant height; *PB* total plant biomass; *SB* shoot biomass; *RB* root biomass; *RS* root to shoot biomass ratio; *GY* grain yield; *HI* harvest index; *rg* genotypic correlation for grain yield

**Table 9:** Phenotypic direct effect (bold diagonal) and indirect effect (off-diagonal) of eight agronomic traits on grain yield of 50 sorghum genotypes

Trait	DTH	DTM	PH	PB	SB	RB	RS	HI	rph
DTH	**0.131**	-0.005	-0.020	-0.002	0.029	-0.004	0.033	0.144	0.306
DTM	-0.015	**0.040**	0.016	0.007	-0.014	-0.062	0.082	-0.134	-0.080
PH	0.071	-0.017	**-0.037**	-0.008	0.068	0.029	0.031	0.130	0.267
PB	0.006	-0.006	-0.007	**-0.043**	0.303	0.224	-0.020	-0.440	0.015
SB	0.010	-0.001	-0.006	-0.033	**0.393**	0.035	0.229	-0.658	-0.033
RB	-0.002	-0.008	-0.003	-0.031	0.045	**0.310**	-0.281	0.030	0.060
RS	-0.010	-0.008	0.003	-0.002	-0.215	0.209	**-0.418**	0.421	-0.021
HI	0.017	-0.005	-0.004	0.017	-0.228	0.008	-0.155	**1.136**	0.786

*DTH* days to 50% heading; *DTM* days to 50% maturity; *PH* plant height; *PB* total plant biomass; *SB* shoot biomass; *RB* root biomass; *RS* root to shoot biomass ratio; *GY* grain yield; *HI* harvest index; *rph* phenotypic correlation for grain yield

## Conclusion

The current study examined the genetic variation and associations among agronomic and carbon storage traits in sorghum genotypes and revealed a considerable genetic variation among the test genotypes. The findings suggest the opportunity for selection and breeding programs to develop improved sorghum cultivars for enhanced yield production and carbon sequestration. The following traits were highly heritable with higher genetic advance values: PB, RB, GY, HI, RS, GCs, RCs, and RCs/SCs, making them ideal traits for selection. The path coefficient analysis revealed that most traits included in the path analysis displayed a positive genotypic and phenotypic direct effect, suggesting direct selection through these traits would be effective for improving grain yield. The study revealed that PB, SB, RB, RS, RCs, and RCs/SCs are the principal traits when selecting sorghum genotypes with high yield and carbon storage capacity.

## Electronic supplementary material

Below is the link to the electronic supplementary material.

Supplementary Material 1

## Data Availability

The datasets generated and analyzed during the current study are available from the corresponding author on reasonable request.
